# Clinical, Laboratory and Instrumental Characteristics of Myocardial Infarction in Young Patients Depending on the Prevalence of Coronary Atherosclerosis

**DOI:** 10.3390/medicina61111996

**Published:** 2025-11-07

**Authors:** Gleb Vladimirovich Nozhov, Kristina Gennadievna Pereverzeva, Sergey Sergeevich Zagorodniy, Sergey Stepanovich Yakushin, Elizaveta Sergeevna Platonova, Angelina Vladimirovna Sermavbrina, German Maksimovich Popov, Elizaveta Romanovna Martynova

**Affiliations:** 1 Department of Hospital Therapy, Ryazan State Medical University, 390026 Ryazan, Russia; glebnozhov@yandex.ru (G.V.N.);; 2 State Budgetary Institution of the Ryazan Region “Regional Clinical Cardiology Dispensary”, 390026 Ryazan, Russia

**Keywords:** myocardial infarction, young age, risk factors, coronary artery disease, dyslipidemia, reperfusion therapy

## Abstract

*Background and Objectives*: To study the clinical/anamnestic, laboratory and instrumental characteristics, as well as the tactics of treatment, of myocardial infarction (MI) in young patients, depending on the number of affected coronary arteries (CAs), including patients with non-ST elevation myocardial infarction. *Materials and Methods*: A single-center retrospective study was conducted based on data from 374 patients under 44 years of age who had experienced myocardial infarction (MI) between 2015 and 2023. The patients were divided into groups according to coronary angiography findings: those without obstructive lesion and those with single-vessel, two-vessel, and multi-vessel disease. Standard methods of statistical analysis were applied. *Results*: A pronounced predominance of men (91.2%) and a high prevalence of modifiable risk factors were identified. The most frequent finding was single-vessel disease (41.9%); however, a significant proportion of patients had two-vessel (25.7%) and multi-vessel (18.2%) CA disease. MI without obstructive CA lesions was diagnosed in 4.3% of patients. Patients with multi-vessel disease had statistically significantly higher levels of total cholesterol and low-density lipoproteins, as well as signs of more pronounced structural cardiac remodeling. *Conclusions*: MI at a young age is associated with a high prevalence of modifiable risk factors and a significant proportion of extensive atherosclerosis. The identification of a group without obstructive CA lesions (4.3%) underscores the heterogeneity of myocardial infarction pathogenesis in the young and the need to account for it in the diagnostic algorithm. The obtained data confirm the necessity of enhancing primary and secondary prevention programs.

## 1. Introduction

Circulatory system diseases remain the leading cause of mortality in the Russian Federation, accounting for over 40% of the structure of overall population mortality. Among circulatory system diseases, coronary artery disease (CAD) holds a special place, accounting for 42.3% of cases (data for 2022) [1]. Myocardial infarction (MI), as one of the most severe manifestations of CAD, is traditionally associated with older patients. However, recent decades have seen a steady increase in MI incidence among young people under 45 years of age, which requires special attention from the medical community [2]. Numerous studies have proven the key role of traditional risk factors (RFs) (smoking, arterial hypertension, dyslipidemia, heredity) in the development of MI in young patients [2,3,4]. The number of affected coronary arteries (CA) is also an important prognostic factor, determining not only the immediate and long-term prognosis but also influencing the choice of revascularization (percutaneous coronary intervention (PCI) or coronary artery bypass grafting) and the intensity of secondary drug prevention [5,6]. At the same time, a significant proportion of young patients with MI do not show hemodynamically significant stenosis on angiography—a phenomenon known as myocardial infarction with non-obstructive coronary arteries (MINOCA), whose pathogenesis and optimal management tactics remain a subject of active study.

Thus, a detailed characterization of young patients with MI depending on the prevalence of coronary atherosclerosis, including the identification of a MINOCA group, appears clinically relevant. The aim of this study was a comparative investigation of clinical-anamnestic, laboratory, and instrumental characteristics, as well as management tactics for MI in young patients depending on the number of affected CAs according to coronary angiography (CAG).

## 2. Materials and Methods

A single-center registry-based retrospective study was conducted at the Ryazan Regional Clinical Cardiological Dispensary. The study included 374 young patients who had experienced MI at the age of 44 years or younger between 1 January 2015, and 31 December 2023. All data were obtained through a retrospective analysis of medical records. All patients provided signed informed voluntary consent for the use of their personal data during hospitalization. The inclusion criterion was age at the time of a verified MI (according to the Fourth Universal Definition of Myocardial Infarction criteria [7]) of ≤44 years. The sole exclusion criterion was the patient’s refusal to sign the voluntary informed consent for the use of personal data. All patients who met the inclusion criteria between 2015 and 2023 were included in the analysis (n = 374). Due to the retrospective design of the study, a formal a priori sample size calculation was not performed. A power analysis for detecting a small effect size at a significance level of α = 0.05 showed that the achieved power for the total sample size of n = 337 exceeded 80%.

For stratification of patients based on the nature of CA involvement, data from CAG, which was performed on 337 (90.1%) patients, were used. A CA lesion was defined as the presence of a hemodynamically significant stenosis (≥50% lumen obstruction). The diameter of the affected artery was not considered, as patient stratification was based on a qualitative assessment of the prevalence of the atherosclerotic process (number of affected vessels), rather than a quantitative assessment of its functional significance. This approach aligns with the study’s objective—to analyze the association between systemic risk factors and the anatomical prevalence of coronary artery involvement. The interpretation of coronary angiograms was performed by two independent cardiologists who were blinded to the clinical and laboratory data of the patients. In case of discrepancies in assessment, a final decision was made with the involvement of a senior expert. Based on the CAG data, these patients were divided into 4 groups: (1) no CA lesion (MINOCA, n = 16), (2) single-vessel disease (n = 157), (3) two-vessel disease (n = 96), 4) three or more CA involvement (multi-vessel disease, n = 68). Data from all 374 included patients were used for the analysis of overall clinical and anamnestic characteristics and management tactics.

Statistical analysis was performed using the StatTech v. 4.8.5 software (developer: StatTech LLC, Kazan, Russia). Comparison of three or more groups on a quantitative measure that did not follow a normal distribution was performed using the Kruskal–Wallis test, with post hoc comparisons using Dunn’s test with the Holm correction. Comparison of percentages in multi-way contingency tables was performed using Pearson’s chi-square test. Post hoc comparisons were performed using Pearson’s chi-square test with the Holm correction. Quantitative measures were assessed for normality using the Kolmogorov–Smirnov test. Data in the tables are presented as median (Me) and interquartile range (IQR). Categorical data are described with absolute values and percentages.

Differences were considered statistically significant at *p* < 0.05.

The study was approved by the Local Ethics Committee of the Federal State Budgetary Educational Institution of Higher Education “Ryazan State Medical University” of the Ministry of Health of the Russian Federation (Protocol No. 7, dated 15 January 2024).

## 3. Results

Among the included patients, 341 (91.2%) were men, and the median age of all included patients was 41 [37, 43] years.

Table 1 presents the clinical and anamnestic characteristics of the patients depending on the number of affected vessels according to CAG.

The most common RFs were male sex (n = 341, 91.2%), overweight and obesity (n = 297, 79.4%), which was classified according to the World Health Organization classification: BMI 25–30 kg/m^2^—pre-obesity, BMI 30–35 kg/m^2^—obesity class I, BMI 35–40 kg/m^2^—obesity class II, and BMI ≥40 kg/m^2^—obesity class III, smoking (n = 290, 77.5%), and a history of hypertension (n = 277, 74.1%). Patients with single-vessel CA disease smoked statistically significantly more often compared to patients without CA lesions (82.2% vs. 50%, *p* = 0.015). The prevalence of other RFs did not differ statistically significantly between the analyzed groups.

Among all included patients, a history of coronary artery disease was present in 50 (13.4%), specifically in 13 (8.3%) in Group 2, 21 (21.9%) in Group 3, and 10 (14.7%) in Group 4 (*p* = 0.013 for the comparison between Groups 2 and 3). There were no such patients in Group 1.

**Figure 1:**
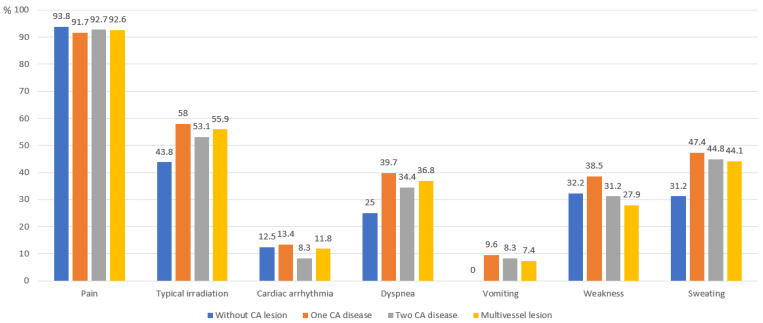
Distribution of complaints upon admission among different patient groups. CA—coronary artery.

The distribution of presenting complaints varied between groups with different numbers of affected vessels (Figure 1). In the group without CA lesions, its frequency reached 93.8%. Typical pain radiation was observed in more than half of the patients with CA disease (58% in Group 2, 53.2% in Group 3, 55.9% in Group 4).

The data of the physical examination in the emergency department, including respiratory rate (RR), systolic (SBP) and diastolic (DBP) blood pressure and heart rate (HR), are presented in Table 2.

Table 3 shows the results of laboratory tests depending on the number of affected CA.

Statistical analysis revealed a number of significant differences. Thus, the level of total cholesterol (TC) was statistically significantly higher in the multi-vessel disease group compared to the group without CA lesions (5.70 [5.04; 6.51] mmol/L vs. 4.65 [3.68; 5.21] mmol/L; *p* = 0.003). Similar data were observed for low-density lipoproteins (LDL): their level progressively increased from the no lesion group (2.57 [1.92; 2.79] mmol/L) to the single-vessel (3.13 [2.48; 3.80] mmol/L; *p* = 0.021), two-vessel (3.12 [2.41; 3.83] mmol/L; *p* = 0.039), and multi-vessel (3.33 [2.62; 4.29] mmol/L; *p* = 0.021) disease groups.

Analysis of myocardial damage and inflammation markers revealed significant differences. The maximum creatine phosphokinase-MB (CK-MB) level was statistically significantly higher in the groups with atherosclerotic lesions: in single-vessel disease (177.72 [51.00; 328.50] U/L, *p* = 0.039), two-vessel disease (157.00 [54.50; 469.50] U/L, *p* = 0.022), and multi-vessel disease (143.00 [66.50; 341.00] U/L, *p* = 0.039) compared to the group without CA lesions (48.00 [28.60; 131.38] U/L). Furthermore, patients with single- and two-vessel disease had a higher leukocyte count (13.00 [10.20; 16.20] × 10^9^/L, *p* = 0.010 and 12.20 [9.85; 15.94] × 10^9^/L, *p* = 0.037, respectively) compared to the MINOCA group (9.73 [7.77; 12.50] × 10^9^/L).

Echocardiography (EchoCG) results are presented in Table 4. The median left ventricular ejection fraction (LVEF) was 52% [47.0–60.0].

Analysis of echocardiography data revealed statistically significant differences in left ventricular (LV) dimensions between the groups. It was found that patients with two-vessel CA disease had a significantly larger LV internal diameter end diastole (LVIDd) (5.50 [5.20; 5.80] cm) compared to patients with single-vessel disease (5.30 [5.00; 5.60] cm; *p* = 0.019). A similar pattern was observed for the LV internal diameter end systole (LVIDs), which was also larger in the two-vessel disease group (3.90 [3.62; 4.28] cm) compared to the single-vessel disease group (3.70 [3.50; 4.00] cm; *p* = 0.035).

**Figure 2:**
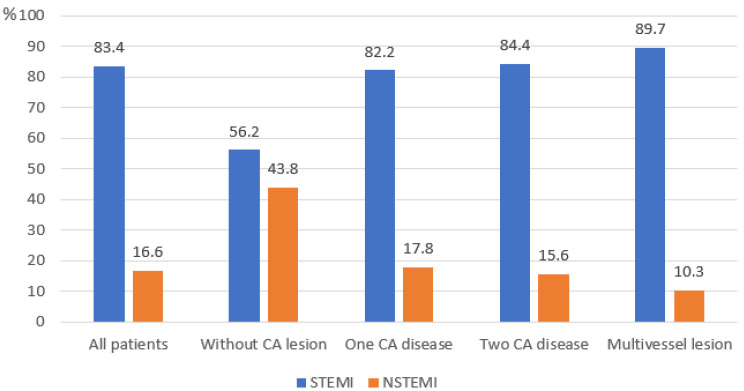
Proportion of patients with STEMI and NSTEMI. STEMI—ST-Elevation Myocardial Infarction, NSTEMI—Non-ST-Elevation Myocardial Infarction, and CA—coronary artery.

The proportion of patients with ST-segment elevation myocardial infarction (STEMI) and non-ST-segment elevation myocardial infarction (NSTEMI) in the patient groups is presented in Figure 2. The extremely high prevalence of STEMI in the entire cohort (83.4%) is noteworthy; it was highest in the multi-vessel disease group (89.7%) and lowest in the MINOCA group (56.2%).

Among patients with STEMI, CAG was performed in 280 cases (89.7%). Among patients with NSTEMI, it was performed in 57 cases (91.9%). Reperfusion tactics for the patients are presented in Table 5.

No statistically significant differences were identified in the analysis of prescribed drug therapy.

In Group 1, 100% of patients received beta-blockers (BBs), statins, and dual antiplatelet therapy (DAPT); 93.4% received angiotensin-converting enzyme inhibitors (ACEIs), and 6.3% received angiotensin II receptor blockers (ARBs).

In Group 2, 93.6% of patients received BBs, 90.5% received ACEIs, 5.1% were prescribed ARBs, and 98.7% received statins. Only 2 patients did not receive DAPT.

In Group 3, 95.8% of patients were prescribed BBs, 94.8% received ACEIs, 4.9% received ARBs, and 98.9% received statins. All patients received DAPT.

In Group 4, 94.1% of patients received BBs, 89.7% received ACEIs, 10.3% received ARBs, and 100% were prescribed statins. DAPT was also prescribed to all patients in this group.

Group 1, consisting of 16 patients (4.3%), was identified as a separate MINOCA category. Patients in this group had a comparable median age (40.0 [36.0; 41.0] years) with the other groups (*p* = 0.282); however, their risk factor profile showed certain specific features. Thus, the proportion of smokers in this group (50.0%) was statistically significantly lower than in the single-vessel disease group (82.2%; *p* < 0.05). Due to the retrospective study design, data on the use of additional imaging modalities (such as intravascular imaging or cardiovascular magnetic resonance) to clarify the pathogenesis of MINOCA were unavailable. Analysis of the clinical presentation revealed that 93.8% of patients in this group presented with typical chest pain. Among patients who underwent CAG, the proportion of those with STEMI in the group without obstructive CA lesions was 56.2%, and with NSTEMI was 43.8%. Regarding reperfusion strategy for STEMI patients in this group, thrombolysis was predominant (33.3%), whereas percutaneous coronary intervention (PCI) was not performed in any case. All patients in Group 1 received comprehensive drug therapy, including BBs, statins, and DAPT. Within the framework of this retrospective study, data on the performance of additional investigations to clarify the mechanism of MINOCA were unavailable.

## 4. Discussion

The demographic and clinical-anamnestic characteristics of the patients are consistent with data from contemporary studies, in which the “rejuvenation” of CAD is associated with a dominance of specific RFs [8,9]. In our study, men were overwhelmingly predominant (91.2%), which aligns with data from other Russian and international registries, where the proportion of men among young patients with MI reaches 70–95% [10,11,12]. The high prevalence of smoking (77.5%), overweight, and obesity (79.4%) underscores the role of modifiable risk factors in the development of premature atherosclerosis, which was widespread in 43.9% of patients, and its acute complications in young individuals. This is consistent with the findings of studies indicating that smoking is a key independent RF for MI in the young [13].

We identified patterns that require explanation. First, the absence of a statistically significant difference in the prevalence of smoking depending on the number of affected vessels (82.2% in single-vessel disease vs. 76.5% in multi-vessel disease, *p* > 0.05). Our data are fully consistent with the results of a recent large study, which also showed that young smokers with ACS have a significantly higher prevalence of single-vessel CA disease [14]. This supports the hypothesis that in young individuals, smoking acts as a powerful trigger for acute coronary events, provoking thrombosis against a background of minimal or moderate atherosclerotic burden. At the same time, the development of extensive multi-vessel atherosclerosis is primarily influenced by other systemic risk factors, such as dyslipidemia, which was also observed in our study in the corresponding patient subgroup [15]. Second, the highest percentage of known CAD history in the two-vessel disease group (21.9% vs. 14.7% in the three-vessel disease group) is also not immediately obvious. We hypothesize that this may be because patients with an established CAD diagnosis had already initiated therapy, which may have slowed the progression of atherosclerosis and prevented its transition to a three-vessel stage, whereas in patients without a prior diagnosis, the disease debuted with extensive involvement from the outset. There is compelling evidence that long-term statin therapy not only reduces lipid levels but also stabilizes atherosclerotic plaques, slowing the overall progression of coronary atherosclerosis [16].

The CAG data are of particular interest. Single-vessel CA disease was the most common finding in young patients (41.9%). However, the significant proportion of patients with two-vessel (25.7%) and multi-vessel (18.2%) disease, as well as the group without hemodynamically significant stenoses (4.3%), indicate the heterogeneity of MI pathogenesis in young adults. Other authors describe a similar distribution; for instance, Ponomarenko I.V. and co-authors report that single-vessel disease was found in 56.5% of patients, two-vessel disease in 21.4%, multi-vessel disease in 10%, and 6.6% of patients had no obstructive CA lesions [3].

Analysis of laboratory parameters revealed statistically significantly higher maximum creatine phosphokinase-MB (CPK-MB) values in the groups with multi-vessel disease, which may indicate more extensive myocardial damage in the presence of multifocal atherosclerosis. Similar data have been obtained in other studies [7]. The presence of dyslipidemia is also noteworthy: median levels of TC and LDL in all groups exceeded target levels, with the most unfavorable lipid profile observed in the group with three or more affected arteries, which is consistent with other research in this field [17]. The progressive worsening of the lipid profile with an increasing number of affected arteries has a clear pathophysiological explanation. Higher and more sustained levels of circulating LDL cholesterol lead to enhanced penetration and retention of apolipoprotein B-containing lipoproteins in the subendothelial space of the coronary arteries [18]. Thus, in patients with multi-vessel disease, we observe the clinical manifestation of this pathological process: persistently elevated levels of atherogenic lipids act as the main “driver” of atherosclerosis progression, leading to the formation of multiple and, likely, more unstable lesions across different vascular beds [19]. This underscores the critical importance of early dyslipidemia control in young individuals.

The EchoCG results showed that the median LVEF remained at a relatively favorable level (52%) in the entire cohort. However, the high prevalence of hypo- and akinesia zones, especially in the groups with multi-vessel disease, indicates significant structural cardiac remodeling after MI.

An important finding of our study is also the exceptionally high proportion of STEMI (83.4%) among young patients, which exceeds the rates typically reported for both the general population [20,21] and young patients [22]. This may serve as a marker of a particularly aggressive disease course in this cohort.

The management and secondary prevention strategies generally aligned with contemporary guidelines, with a high percentage of PCI performed and evidence-based drug therapy (DAPT, statins, BBs, ACEIs/ARBs) prescribed across all groups [1]. The failure to prescribe prognosis-improving medications in individual patients was likely associated with individual contraindications and tolerability.

The group of patients with MINOCA is of particular interest, with a prevalence of 4.3% in our study. This figure is lower than the 5–15% range typically reported in similar studies [23,24,25,26] and is lower than, for example, in the ORPKI registry (7.8%), where MINOCA patients were also younger [27]. Possible explanations for the relatively low frequency include population-specific characteristics and the specifics of the patient referral pathway, whereby some MINOCA cases might not have reached the cardiology dispensary and were instead directed to cardiology or therapeutic departments of other medical institutions. Despite the absence of obstructive atherosclerosis, the risk factor profile in these patients, except for a lower prevalence of smoking, was comparable to the other groups. The high frequency of typical chest pain and the significant proportion of STEMI underscore that MINOCA is an acute and severe condition. The retrospective design of the study did not allow for the determination of the pathogenetic mechanisms of MI in this group, which requires targeted, in-depth investigation. The identification and correct stratification of MINOCA patients are critically important, as their pathogenesis, prognosis, and consequently, optimal secondary prevention strategies fundamentally differ from those in patients with atherothrombosis. This area appears extremely promising for further prospective research. Consequently, a logical continuation of this work is a prospective assessment of survival and the frequency of reaching endpoints in this cohort, which will determine the prognostic significance of the identified angiographic and clinical phenotypes (such as MINOCA and multi-vessel disease) in the long-term period.

The present study has several limitations that should be considered when interpreting the results. First, the single-center retrospective design cannot fully exclude the influence of unaccounted factors. Second, modern imaging methods were not used to assess plaque morphology in patients with atherosclerosis. Third, for patients with MINOCA, data from cardiovascular magnetic resonance imaging or intravascular imaging were unavailable, which precluded the determination of the precise pathogenesis of myocardial infarction in this subgroup. Furthermore, there are no data on long-term follow-up, clinical outcomes, treatment adherence, or socio-economic factors. Prospective multicenter studies are required to overcome these limitations and obtain a more comprehensive picture.

## 5. Conclusions

This study on MI in young patients in Russia allows us to draw the following main conclusions:

1. MI in young Russians is characterized by an extremely high prevalence of modifiable risk factors (smoking, obesity, dyslipidemia), a significant proportion of extensive atherosclerosis (43.9%), and a high frequency of STEMI (83.4%). However, this high prevalence may also reflect referral bias to a specialized cardiology center, where classic STEMI cases are more frequently admitted. This rate exceeds other studies and requires validation in prospective, population-based cohorts.
2. Alongside atherosclerotic forms, a clinically significant MINOCA subgroup (4.3%) was identified. Its lower prevalence compared to international data requires further investigation.
3. It is necessary to strengthen both primary prevention, aimed at risk factor control among youth, and secondary prevention.
4. Key directions for future work include organizing prospective multicenter studies utilizing modern imaging methods for an in-depth investigation of pathophysiology, including MINOCA, and for assessing long-term outcomes in this patient category.

## Figures and Tables

**Table 1:** Clinical and anamnestic characteristics of patients.

Indicator	All Patients (n = 374)	No CA Lesion (n = 16)	Single-Vessel Disease (n = 157)	Two-Vessel Disease (n = 96)	Multi-Vessel Disease (n = 68)	*p*
Age, years, Me (IQR)	41.0 [37.0; 43.0]	40.0 [36.0; 41.0]	41.0 [37.0; 43.0]	41.0 [37.8; 44.0]	41.0 [38.0; 43.0]	0.282
Male, n (%)	341 (91.2)	15 (93.8)	141 (89.8)	90 (93.8)	63 (92.6)	0.697
Pre-obesity, n (%)	159 (42.5)	7 (43.8)	70 (44.6)	33 (34.4)	35 (51.5)	0.052
Obesity class I, n (%)	95 (25.4)	1 (6.2)	36 (22.9)	26 (27.1)	21 (30.9)	
Obesity class II, n (%)	34 (9.1)	1 (6.2)	20 (12.7)	9 (9.4)	3 (4.4)	
Obesity class III, n (%)	9 (2.4)	0 (0.0)	3 (1.9)	3 (3.1)	2 (2.9)	
Smoking, n (%)	290 (77.5)	8 (50.0)	129 (82.2)	73 (76.0)	52 (76.5)	0.028 p_1–2_ = 0.015
Burdened heredity ^1^, n (%)	172 (46.0)	4 (25.0)	68 (43.3)	46 (47.9)	34 (50.0)	0.286
Arterial hypertension, n (%)	277 (74.1)	12 (75.0)	116 (73.9)	72 (75.0)	55 (80.9)	0.729
Diabetes mellitus or impaired glucose tolerance, n (%)	69 (18.4)	1 (6.2)	26 (16.6)	20 (20.8)	17 (25.0)	0.251
STEMI ^2^, n (%)	312 (83.4)	9 (56.2)	129 (82.2)	81 (84.4)	61 (89.7)	0.015 p_1–3_ = 0.044 p_1–4_ = 0.007
NSTEMI ^3^, n (%)	62 (16.6)	7 (43.8)	28 (17.8)	15 (15.6)	7 (10.3)	

^1^—Presence of cardiovascular diseases in the medical history of the mother or sisters under 65 years of age; or of the father or brothers under 55 years of age. ^2^—ST-Elevation Myocardial Infarction, ^3^—Non-ST-Elevation Myocardial Infarction.

**Table 2:** Physical examination data for patients in the emergency department.

Indicator	All Patients (n = 374)	No CA Lesion (n = 16)	Single-Vessel Disease (n = 157)	Two-Vessel Disease (n = 96)	Multi-Vessel Disease (n = 68)	*p*
RR ^1^, breath per min	16.0 [16.0; 17.0]	17.0 [16.0; 17.0]	16.0 [16.0; 17.0]	16.0 [16.0; 17.0]	16.0 [16.0; 18.0]	0.326
SBP ^2^ at admission, mmHg.	132.0 [120.0; 150.0]	140.0 [130.0; 162.5]	130.0 [120.0; 150.0]	140.0 [120.0; 150.0]	140.0 [128.8; 150.0]	0.536
DBP ^3^ at admission, mmHg.	80.0 [80.0; 90.0]	82.5 [80.0; 92.5]	80.0 [80.0; 90.0]	84.5 [80.0; 90.0]	90.0 [80.0; 90.0]	0.545
HR ^4^, bpm	78.0 [70.0; 89.0]	85.5 [73.0; 90.5]	76.0 [67.0; 85.0]	80.0 [72.0; 90.0]	78.0 [70.0; 88.0]	0.006 p_2–3_ = 0.012

^1^—respiratory rate, ^2^—systolic blood pressure, ^3^—diastolic blood pressure, ^4^—heart rate.

**Table 3:** Laboratory test results in the included patients.

Indicator	All Patients (n = 374)	No CA Lesion (n = 16)	Single-Vessel Disease (n = 157)	Two-Vessel Disease (n = 96)	Multi-Vessel Disease (n = 68)	*p*
Hemoglobin max, g/L	155.0 [147.0; 163.3]	150.00 [143.75; 159.50]	155.00 [148.00; 164.00]	154.50 [149.75; 166.00]	157.00 [148.50; 162.00]	0.484
White blood cells max, 10^9^/L	12.3 [9.7; 15.7]	9.73 [7.77; 12.50]	13.00 [10.20; 16.20]	12.20 [9.85; 15.94]	12.07 [9.62; 14.45]	0.010 p_1–2. 1–3_ < 0.05
AST ^1^ max, U/L	86.0 [40.6; 223.7]	66.67 [41.65; 109.12]	84.80 [46.00; 230.80]	108.00 [48.25; 248.00]	106.70 [42.51; 247.50]	0.500
ALT ^2^ max, U/L	56.3 [37.2; 83.9]	60.75 [36.67; 72.55]	56.30 [39.20; 83.30]	60.80 [39.85; 91.19]	51.80 [37.05; 81.64]	0.696
Total bilirubin max, μmol/L	12.5 [8.9; 17.1]	16.34 [12.20; 20.18]	12.20 [8.60; 16.30]	12.50 [8.98; 18.06]	12.70 [9.50; 16.10]	0.171
APTT ^3^, s	31.4 [27.9; 37.7]	32.15 [27.73; 35.77]	31.30 [28.00; 36.45]	31.90 [28.00; 41.60]	31.50 [27.30; 37.00]	0.730
PTI ^4^, %	0.9 [0.9; 1.0]	0.99 [0.88; 1.00]	0.93 [0.88; 1.00]	0.93 [0.88; 1.00]	0.93 [0.87; 1.00]	0.774
Troponin I max, ng/mL	10.7 [1.0; 50.0]	4.68 [1.00; 358.02]	13.80 [1.00; 50.00]	13.40 [1.00; 50.00]	9.16 [1.00; 44.95]	0.788
CK-MB ^5^ max, U/L	132.7 [41.0; 329.9]	48.00 [28.60; 131.38]	177.72 [51.00; 328.50]	157.00 [54.50; 469.50]	143.00 [66.50; 341.00]	0.036 p_1–2. 1–3. 1–4_ < 0.05
Fasting glucose max, mmol/L	5.8 [5.1; 6.8]	6.05 [5.40; 6.90]	5.68 [5.10; 6.74]	6.03 [5.30; 6.89]	5.86 [5.12; 6.79]	0.233
Creatinine max, μmol/L	94.0 [83.0; 105,1]	101.25 [78.17; 116.15]	96.00 [81.50; 103.30]	94.00 [82.50; 105.85]	93.00 [85.50; 102.70]	0.958
TC ^6^ max, mmol/L	5.3 [4.5; 6.2]	4.65 [3.68; 5.21]	5.29 [4.51; 6.19]	5.32 [4.37; 6.37]	5.70 [5.04; 6.51]	0.004 p_1–4_ = 0.003
LDL ^7^ max, mmol/L	3.1 [2.4; 3.8]	2.57 [1.92; 2.79]	3.13 [2.48; 3.80]	3.12 [2.41; 3.83]	3.33 [2.62; 4.29]	0.005 p_1–2. 1–3. 1–4_ < 0.05
HDL ^8^ max, mmol/L	1.1 [0.9; 1.3]	1.15 [1.02; 1.48]	1.04 [0.89; 1.25]	1.12 [0.90; 1.34]	1.03 [0.90; 1.21]	0.292
TG ^9^ max, mmol/L	1.8 [1.1; 2.5]	1.68 [0.82; 2.50]	1.85 [1.15; 2.75]	1.55 [1.07; 2.23]	1.88 [1.27; 2.30]	0.286

^1^—Aspartate aminotransferase, ^2^—Alanine aminotransferase, ^3^—activated partial thromboplastin time, ^4^—the prothrombin index, ^5^—creatine phosphokinase-MB, ^6^—total cholesterol, ^7^—low-density lipoproteins, ^8^—high-density lipoproteins, ^9^—triglycerides.

**Table 4:** Results of laboratory tests in patients who underwent MI at a young age, depending on the number of affected CA.

Indicator	All Patients (n = 374)	No CA Lesion (n = 16)	Single-Vessel Disease (n = 157)	Two-Vessel Disease (n = 96)	Multi-Vessel Disease (n = 68)	*p*
LVIDd ^1^, Me [IQR]	5.4 [5.1; 5.7]	5.54 [5.28; 5.63]	5.30 [5.00; 5.60]	5.50 [5.20; 5.80]	5.50 [5.10; 5.70]	0.010 p_2–3_ = 0.019
LVIDs ^2^, Me [IQR]	3.8 [3.6; 4.2]	3.84 [3.60; 4.24]	3.70 [3.50; 4.00]	3.90 [3.62; 4.28]	3.90 [3.60; 4.22]	0.018 p_2–3_ = 0.035
LA ^3^, Me [IQR]	3.9 [3.6; 4.1]	3.90 [3.50; 3.98]	3.85 [3.60; 4.10]	4.00 [3.60; 4.13]	3.90 [3.65; 4.14]	0.623
Interventricular septum thickness, Me [IQR]	1.1 [1.0; 1.2]	1.00 [0.97; 1.10]	1.06 [1.00; 1.18]	1.10 [0.98; 1.19]	1.08 [1.00; 1.17]	0.646
LVPWT ^4^, Me [IQR]	1.0 [0.9; 1.1]	1.00 [0.90; 1.00]	1.00 [0.90; 1.02]	1.00 [0.90; 1.10]	1.00 [0.90; 1.08]	0.782
RV ^5^, Me [IQR]	2.5 [2.3; 2.7]	2.55 [2.20; 2.79]	2.50 [2.40; 2.70]	2.50 [2.30; 2.68]	2.50 [2.31; 2.60]	0.795
LVEF ^6^, Me [IQR]	52.0 [47.0; 60.0]	54.50 [50.00; 61.25]	53.00 [48.00; 60.00]	51.00 [46.25; 57.00]	52.00 [48.00; 58.50]	0.195
LVEF, abs. (%)						0.407
-LVEF ≤ 50%	150 (41.1)	6 (37.5)	56 (36.4)	44 (46.8)	29 (43.3)	
-LVEF > 50%	214 (58.9)	10 (62.5)	98 (63.6)	50 (53.2)	38 (56.7)	
Akinesia zones, abs. (%)	146 (40)	2 (12.5%)	60 (39.0%)	40 (42.6%)	23 (34.3%)	0.128
Hypokinesia zones, abs. (%)	240 (65.8)	15 (93.8%)	93 (60.4%)	62 (66.0%)	49 (73.1%)	0.027 p_1–2_ = 0.050

^1^—left ventricle internal diameter end diastole, ^2^—left ventricle internal diameter end systole, ^3^—left atrium, ^4^—c, ^5^—right ventricle, ^6^—left ventricular ejection fraction.

**Table 5:** Proportion of patients undergoing reperfusion treatment in the analyzed patient groups.

STEMI ^1^						
Indicator	All patients (n = 280)	No CA Lesion (n = 9)	Single-Vessel Disease (n = 129)	Two-Vessel Disease (n = 81)	Multi-Vessel Disease (n = 61)	*p*
Thrombolysis, abs (%)	10 (3.6)	3 (33.3)	5 (3.9)	0 (0.0)	2 (3.3)	<0.001 p_1–2. 1–3. 1–4_ < 0.05
PTCA & CAS ^2^, abs. (%)	179 (63.9)	0 (0.0)	78 (60.5)	58 (71.6)	43 (70.5)	<0.001 p_1–2. 1–3. 1–4_ < 0.05
Pharmaco-invasive strategy, abs. (%)	74 (26.4)	0 (0.0)	40 (31.0)	20 (24.7)	14 (23.0)	0.163
Without reperfusion, abs. (%)	17 (6.1)	6 (66.7)	6 (4.7)	3 (3.7)	2 (3.3)	<0.001 p_1–2. 1–3. 1–4_ < 0.05
**NSTEMI** **^3^**						
**Indicator**	**All patients** **(n = 57)**	**No CA Lesion** **(n = 7)**	**Single-Vessel Disease** **(n = 28)**	**Two-Vessel Disease** **(n = 15)**	**Multi-Vessel Disease** **(n = 7)**	***p***
PTCA & CAS, aбc. (%)	44 (77.2)	0 (0.0)	23 (82.1)	15 (100.0)	6 (85.7)	<0.001 p_1–2. 1–3. 1–4_ < 0.05
Without reperfusion, abs. (%)	13 (22.8)	7 (100.0)	5 (17.9)	0 (0.0)	1 (14.3)	<0.001 p_1–2. 1–3. 1–4_ < 0.05

^1^—ST-Elevation Myocardial Infarction, ^2^—percutaneous transluminal coronary angioplasty and coronary artery stenting, ^3^—Non-ST-Elevation Myocardial Infarction.

## Data Availability

The data presented in this study is only available upon request from the relevant author due to the privacy of the data in the medical records.

## Abbreviations

The following abbreviations are used in this manuscript:

MI	Myocardial infarction
RF	Risk factors
CA	Coronary artery
CAD	Coronary artery disease
PCI	Percutaneous coronary intervention
MINOCA	Myocardial infarction with non-obstructive coronary arteries
CAG	Coronary angiography
RR	Respiratory rate
SBP	Systolic blood pressure
DBP	Diastolic blood pressure
HR	Heart rate
AST	Aspartate aminotransferase
ALT	Alanine aminotransferase
APTT	Activated partial thromboplastin time
PTI	The prothrombin index
CK-MB	Creatine phosphokinase-MB
TC	Total cholesterol
LDL	Low-density lipoproteins
HDL	High-density lipoproteins
TG	Triglycerides
EchoCG	Echocardiography
LVEF	Left ventricle ejection fraction
LVIDd	Left ventricle diastolic dimension
LVIDs	Left ventricle systolic dimension
LA	Left atrium
LVPWT	Left ventricular posterior wall thickness
RV	Right ventricle
LV	Left ventricle
STEMI	Myocardial infarction with ST segment elevation
NSTEMI	Myocardial infarction without ST segment elevation
PCI & CAS	Percutaneous coronary intervention and coronary artery stenting
BAB	Beta-blockers
ACE inhibitors	Angiotensin-converting enzyme inhibitors
ARBs	Angiotensin II receptor blockers
